# Assessment of risk factors for black triangle formation after orthodontic treatment with premolar extractions

**DOI:** 10.21142/2523-2754-1401-2026-276

**Published:** 2025-12-28

**Authors:** Nazanin Ghasemi, Arezoo Jahanbin, Mona Kazemi, Erfan Shadi, Mostafa Shahabi, Ziba Shirkhani

**Affiliations:** 1 Dentist. Mashhad, Iran. nazaninghassemi80@gmail.com Mashhad Iran nazaninghassemi80@gmail.com; 2 Professor, Department of Orthodontics, School of Dentistry, Mashhad University of Medical Sciences. Mashhad, Iran. jahanbina@mums.ac.ir shahabim@mums.ac.ir Mashhad University of Medical Sciences Department of Orthodontics School of Dentistry Mashhad University of Medical Sciences Mashhad Iran jahanbina@mums.ac.ir shahabim@mums.ac.ir; 3 Resident, Department of Orthodontics, School of Dentistry, Mashhad University of Medical Sciences. Mashhad, Iran. monakazemi.dnt@gmail.com shadie4031@mums.ac.ir Mashhad University of Medical Sciences Department of Orthodontics School of Dentistry Mashhad University of Medical Sciences Mashhad Iran monakazemi.dnt@gmail.com shadie4031@mums.ac.ir; 4 Dental Research Center, Mashhad University of Medical Sciences, Mashhad, Iran. shirkhaniziba2@gmail.com Mashhad University of Medical Sciences Dental Research Center Mashhad University of Medical Sciences Mashhad Iran

**Keywords:** orthodontics, black triangle, risk factors, ortodoncia, triángulo negro, factores de riesgo

## Abstract

**Aim:**

Black triangles (open gingival embrasures) result from a reduction in the height of the interdental papilla below the contact point and can lead to food impaction, alveolar bone loss, difficulties in plaque control, compromised esthetics, and speech problems. Since achieving a harmonious and esthetic smile is one of the primary goals of orthodontic treatment, identifying risk factors associated with these spaces is of great clinical importance. The aim of the present study was to investigate and compare risk factors related to the occurrence of black triangles after orthodontic treatment.

**Methods:**

Records of 47 patients between the ages of 14-34 years old, all treated with fixed orthodontics using the MBT 0.22 system and bilateral premolar extraction, were retrospectively analyzed. Patients were divided into two groups based on the presence (n=25) or absence (n=22) of black triangles after treatment. Pre- and post-treatment panoramic radiographs, lateral cephalograms, and intraoral photographs were calibrated and analyzed using ImageJ and Dolphin Imaging software. Measurements included distance from contact point to alveolar crest, horizontal and vertical incisor movements, tooth morphology, interproximal CEJ distance, axial inclination of incisors, Little’s irregularity index, contact area, and treatment duration. Data were analyzed using paired and independent t-tests, Mann-Whitney U test, and Fisher’s exact test (significance level set lesser than 0.05).

**Results:**

The findings revealed that the distance from the contact point to the alveolar crest showed a statistically significant difference between the two groups in the mandibular arch (p = 0.006) and in the total of both arches combined (p = 0.003). However, the difference in the maxillary arch was not statistically significant (p = 0.127). Moreover, in patients without black triangles, the mean distance from the contact point to the alveolar crest showed a slight, nonsignificant decrease in both arches(P>0.05). In contrast, patients with black triangles exhibited no significant maxillary change but a significant mandibular increase from 3.44 mm pre- treatment to 4.19 mm post-treatment (p = 0.021). Other variables, including horizontal and vertical incisor movements, tooth morphology, interproximal CEJ distance, axial inclination, irregularity index, contact area, and treatment duration, did not show statistically significant differences (P>0.05).

**Conclusion:**

Maintaining a short vertical distance between the contact point and the alveolar bone crest plays a critical role in minimizing black triangle formation after orthodontic treatment. When treatment procedures are standardized, variables such as tooth angulation, crown shape, and treatment duration appear to have limited impact. This study demonstrated that patients who developed black triangles showed a significant post-treatment increase in the mandibular contact point to the alveolar crest distance. Recognizing this relationship can help clinicians refine treatment strategies to improve aesthetics and preserve periodontal health.

## RESUMEN

**Objetivo:**

Los triángulos negros (troneras gingivales abiertas) son el resultado de una reducción en la altura de la papila interdental, por debajo del punto de contacto, y pueden provocar impactación de alimentos, pérdida ósea alveolar, dificultades en el control de la placa, compromiso estético y problemas del habla. Dado que lograr una sonrisa armoniosa y estética es uno de los objetivos principales del tratamiento de ortodoncia, identificar los factores de riesgo asociados con estos espacios resulta de gran importancia clínica. El objetivo del presente estudio fue investigar y comparar los factores de riesgo relacionados con la aparición de triángulos negros después del tratamiento de ortodoncia.

**Métodos:**

Se analizaron retrospectivamente los registros de 47 pacientes de entre 14 y 34 años, todos tratados con ortodoncia fija mediante el sistema MBT 0.22 y extracción bilateral de premolares. Los pacientes se dividieron en dos grupos según la presencia (n = 25) o ausencia (n = 22) de triángulos negros después del tratamiento. Las radiografías panorámicas, los cefalogramas laterales y las fotografías intraorales pre y postratamiento se calibraron y analizaron con los programas ImageJ y Dolphin Imaging. Las mediciones incluyeron la distancia desde el punto de contacto hasta la cresta alveolar, los movimientos horizontales y verticales de los incisivos, la morfología dental, la distancia interproximal de la unión alveolar (LAC), la inclinación axial de los incisivos, el índice de irregularidad de Little, el área de contacto y la duración del tratamiento. Los datos se analizaron mediante pruebas T pareadas e independientes, la prueba U de Mann-Whitney y la prueba exacta de Fisher (nivel de significación inferior a 0,05).

**Resultados:**

Los hallazgos revelaron que la distancia desde el punto de contacto hasta la cresta alveolar mostró una diferencia estadísticamente significativa entre los dos grupos en la arcada mandibular (p = 0,006) y en la suma de ambas arcadas (p = 0,003). Sin embargo, la diferencia en la arcada maxilar no fue estadísticamente significativa (p = 0,127). Además, en pacientes sin triángulos negros, la distancia media desde el punto de contacto hasta la cresta alveolar mostró una ligera disminución no significativa en ambas arcadas (p > 0,05). Por el contrario, los pacientes con triángulos negros no mostraron cambios maxilares significativos, pero sí un aumento mandibular significativo de 3,44 mm antes del tratamiento a 4,19 mm después (p = 0,021). Otras variables, como los movimientos horizontales y verticales de los incisivos, la morfología dental, la distancia interproximal de la unión alveolar (LAC), la inclinación axial, el índice de irregularidad, el área de contacto y la duración del tratamiento, no mostraron diferencias estadísticamente significativas (p > 0,05).

**Conclusión:**

Mantener una distancia vertical corta entre el punto de contacto y la cresta ósea alveolar desempeña un papel fundamental para minimizar la formación de triángulos negros después del tratamiento de ortodoncia. Cuando se estandarizan los procedimientos de tratamiento, variables como la angulación dental, la forma de la corona y la duración del tratamiento parecen tener un impacto limitado. Este estudio demostró que los pacientes que desarrollaron triángulos negros mostraron un aumento significativo en la distancia entre el punto de contacto mandibular y la cresta alveolar después del tratamiento. Reconocer esta relación puede ayudar a los profesionales clínicos a refinar las estrategias de tratamiento para mejorar la estética y preservar la salud periodontal.

## INTRODUCTION

Over the past decades, the motivation for seeking orthodontic treatment has shifted significantly. Whereas in the past the primary goal was correction of functional problems and malocclusion, today esthetic concerns are equally important, particularly among adolescents and adults ^1^. This shift reflects socioeconomic development and changing social norms that emphasize dental and facial appearance.

The growing demand for esthetic outcomes introduces new challenges for orthodontists and periodontists. Treatment planning now requires careful evaluation of both dental alignment and periodontal support, and an interdisciplinary approach is often essential to maintain periodontal health while achieving satisfactory esthetic results ^2^.

Orthodontics is more than an esthetic procedure; it improves occlusion, mastication, speech, and even airway function. However, like any medical intervention, orthodontic treatment may have side effects. Reported complications include root resorption, pain, pulpal changes, decalcification, temporomandibular disorders, and periodontal problems ^3^^-^^6^.

One of the most esthetically concerning outcomes is the appearance of black triangles, or open gingival embrasures. These occur when the interdental papilla fails to completely occupy the interproximal space, leaving a triangular void ^7^. Patients often perceive these spaces as highly unattractive, with negative impacts on self-confidence and psychosocial well-being. Functionally, they may lead to food impaction, plaque retention, halitosis, caries, and gingival inflammation ^8^.

The etiology of black triangles is multifactorial. Contributing factors include triangular crown morphology, divergent root angulation, increased distance between contact point and alveolar crest, alveolar bone resorption, periodontal disease, aging, and orthodontic tooth movement ^9^^,^^10^. In some cases, orthodontics can exacerbate the problem, while in others, it can help close embrasures.

Prevalence studies report that 36-43% of adults and approximately 15% of adolescents develop black triangles after orthodontic treatment, with the maxillary anterior region most affected ^11^. The critical determinant is the distance between the interproximal contact point and alveolar crest; papillary fill is predictable when this distance is ≤5 mm but decreases significantly as the distance increases ^12^.

Black triangles present both esthetic and functional challenges. Even small embrasures (<3 mm) are noticeable to patients and dentists. They rank as the third most significant esthetic dental problem after caries and dark crown margins ^13^.

Management of black triangles is complex. Reconstructing lost papilla is unpredictable because of limited vascularity. Available approaches include surgical techniques such as connective tissue grafts, minimally invasive procedures like hyaluronic acid injection, restorative camouflage with composites or veneers, and orthodontic strategies such as interproximal reduction to broaden contact areas ^14^.

Despite some studies ^15^^-^^17^ existing evidence on risk factors for black triangle formation remains inconsistent. Variability in methodology, sample characteristics, and measurement techniques contributes to conflicting results ^15^. Some studies have focused on single factors, whereas the condition likely results from interactions between multiple variables including tooth morphology, axial inclination, CEJ-to-crest distance, and periodontal status. Moreover, heterogeneous or small samples reduce reliability of findings ^16^^,^^17^.

So, the present study seeks to determine the factors influencing the occurrence of black triangles after orthodontic treatment with premolar extractions, with the goal of improving prevention and management strategies in clinical practice.

## MATERIAL AND METHODS

This study was carried out at School of Dentistry of Mashhad University of Medical Science, from September 2023 to March 2024. The ethical code IR.MUMS.DENTISTRY.REC.1403.038 was assigned to this study.

This retrospective longitudinal study included the records of 47 patients (7 males and 40 females) aged 14-34 years, who received orthodontic treatment either at the Orthodontic Department of Mashhad Dental School or a private clinic. Patients were treated with the MBT 0.022-inch bracket system with extraction of premolars in both jaws.

Inclusion criteria for the study were as follows: patients who had completed fixed orthodontic treatment and presented with Class I malocclusion accompanied by crowding. All subjects had undergone extraction of the first or second premolars in both arches as part of their treatment plan. Orthodontic therapy was carried out using the MBT 0.022-inch system. Only patients with complete documentation were included, which comprised patient files, pre- and post-treatment lateral cephalograms, panoramic radiographs (OPG), intraoral and extraoral photographs, and study casts.

Exclusion criteria included patients presenting with complex conditions such as impacted canines, cleft palate, or a history of orthognathic surgery. Cases that had undergone camouflage treatment for Class II or Class III malocclusion were excluded, as were those with periodontal disease or a history of periodontal surgery in the anterior region. Patients with a history of anterior interproximal reduction (stripping) during orthodontic treatment, absence of one or more anterior teeth, or previous orthodontic treatment were not considered. In addition, records with photographs of poor quality or containing artifacts, such as saliva, food remnants, or impression material between the teeth, were excluded from the study.

All patient records were carefully examined. Pre-treatment intraoral photographs were reviewed to ensure the absence of black triangles at the beginning of the study. Post-treatment photographs were then evaluated to determine the presence or absence of black triangles in the anterior interproximal spaces. Based on post-treatment photograph evaluation, patients were classified into two groups:

•Black triangle group: Patients with one or more black triangles in the anterior region from canine to canine in the maxilla or mandible (n = 25).
•No black triangle group: Patients without any visible black triangles in the anterior interproximal spaces (n = 22).

Pre- and post-treatment panoramic (OPG) and lateral cephalometric radiographs were scanned using the radiology scanner. Intraoral photographs were captured with an iPhone 12 Pro.

In panoramic radiographs (OPG) and intraoral photographs, the mesiodistal width of the upper left central incisor was measured on the patient’s study cast using an Orthometric caliper. The same distance was then measured digitally with a JIR caliper (China) with an accuracy of 0.01 mm and applied to calibrate the magnification of OPGs and photographs.

All OPG and photographic images were first converted to JPEG format and imported into ImageJ software (NIH, Bethesda, MD, USA), while cephalograms were analyzed with Dolphin Imaging software version 11 (Dolphin Imaging & Management Solutions, Chatsworth, CA, USA). To set the scale, the Line Tool was selected, and a line was drawn along the incisal edge of the upper left central incisor. The scale was set via Analyze > Set Scale, entering the real tooth length (measured with the digital caliper) as the Known Distance in millimeters and setting the Unit of Length to “mm.” After confirming the settings, the image was calibrated to real-world units, and all subsequent measurements were performed using this scale.

Linear distances were measured using the Line Tool and the Analyze > Measure function, with results automatically displayed in the “Results” window and saved for analysis. Angular measurements on OPGs and photographs were performed using the Angle Tool. Three points were identified: the vertex of the angle and two points along the intersecting lines. After drawing the angle, the Analyze > Measure function provided the angular value in degrees in the Results window.

After each measurement, a new row was added to the results table, and the corresponding values were recorded. All results were saved for documentation and statistical analysis using File > Save As.

### Variables Assessed

**Figure 1:**
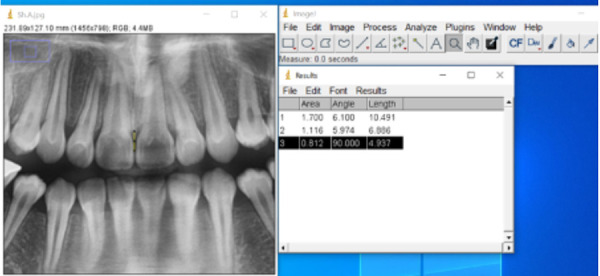
Method for measuring the distance from the contact point to the alveolar crest between two adjacent teeth: In this image, the distance from the contact point to the alveolar crest between the maxillary central incisors is 4.937 mm.

1. Anterior Tooth Retraction/Protrusion: Pre- and post-treatment lateral cephalograms were traced in Dolphin software, and U1-SN, U1-NA, L1-NB, and IMPA were measured.
2. Intrusion/Extrusion of Incisors: The vertical distances from the incisal edges of the upper and lower central incisors to the palatal and mandibular planes, respectively, were measured in millimeters on the cephalograms.
3. Crown Shape: Ratio of maximum mesiodistal crown width to width at the cementoenamel junction (CEJ) measured on OPG images.
4. Contact Point to Alveolar Crest Distance: Distance from the most cervical point of the contact area to the alveolar crest on OPG (Figure 1).
5. Inter-CEJ Distance: Distance between CEJs of adjacent teeth measured on OPG.
6. Axial Angles of Adjacent Teeth: Angle between the long axes of adjacent anterior teeth; divergent roots were assigned negative angles and convergent roots positive angles.
7. Contact Area: Vertical distance between the most apical and coronal points of proximal contact between adjacent teeth.
8. Treatment Duration: Calculated in months from treatment start to end of Orthodontic treatment.
9. Initial Crowding: Measured using Little’s Irregularity Index on pre-treatment occlusal photographs of the maxilla and mandible.

All measurements were recorded and analyzed using SPSS software version 21. Analytical comparisons were performed using independent and paired t-tests, Mann-Whitney, and Fisher’s exact tests, with significance set at p < 0.05.

## RESULTS

In this study, a total of 47 orthodontic patient records were analyzed, including 40 females (85.1%) and 7 males (14.9%), with a mean age of 19.98 ± 3.80 years (range 14-34 years). Variables examined included tooth shape, distance from contact point to alveolar crest, distance of adjacent CEJ after treatment, amount of anterior tooth retraction/protrusion in both jaws, vertical movement of incisors, Little’s irregularity index before treatment, contact area after treatment, and treatment duration, comparing groups with and without black triangles.

Table 1 shows the age distribution for the two groups. The youngest patient without black triangles was 14 years old, and the oldest was 26 years old; for the black triangle group, the youngest was 14 and the oldest was 34. The mean age ± SD for the non-black triangle group was 19.14 ± 2.90 years, and for the black triangle group was 20.72 ± 4.37 years, with no statistically significant difference (P = 0.264).

**Table 1:** Age distribution between groups with and without black triangles

Group	N	Mean ± SD	IQR	Min Max	Mann- Whitney test
Without black triangle	22	19.14 ± 2.90	4	14	0.264=p
				26	
With black triangle	25	20.72 ± 4.37	6	14	
				34	

Table 2 shows sex distribution. The number of females in the black triangle and non-black triangle groups was 23 (92%) and 17 (77.3%), respectively, and the number of males was 2 (8%) and 5 (22.7%), respectively, with no significant difference (P=0.228).

**Table 2:** Sex distribution between groups with and without black triangles

Group		sex		Total
		female	male	
With black triangle	N	23	2	25
	%	92/0%	8/0%	100/0%
With black triangle	N	17	5	22
	%	77/3%	22/7%	100/0%
Total	N	40	7	47
	%	85.1%	14/9%	100/0%
Fisher’s Exact Test	p = 0.228 x^2^ = 2.002					

Table 3 shows the distribution of black triangles in anterior teeth. The most common location was in the upper midline and lower left central-lateral area.

**Table 3:** Distribution of black triangles in anterior teeth

	UL3,2	UL2,1	U1,1	UR2,1	UR3,2
Number of black triangles	0	3	9	6	1
Percent of black triangles	0	6.4	19.1	12.8	2.1
	LL3,2	LL2,1	L1,1	LR2,1	LR3,2
Number of black triangles	5	15	11	13	6
Percent of black triangles	10.6	31.9	23.4	27.7	12.8

UR3,2: between upper right canine and upper right lateral incisor

UR2,1: between upper right lateral incisor and upper right central incisor

U1,1: between upper right central incisor and upper left central incisor (midline)

UL2,1: between upper left lateral incisor and upper left central incisor

UL3,2: between upper left canine and upper left lateral incisor

LR3,2: between lower right canine and lower right lateral incisor

LR2,1: between lower right lateral incisor and lower right central incisor

L1,1: between lower right central incisor and lower left central incisor (midline)

LL2,1: between lower left lateral incisor and lower left central incisor

LL3,2: between lower left canine and lower left lateral incisor

Table 4 summarizes U1_SN, U1_NA, IMPA, and L1_NB changes before and after treatment and their differences. No statistically significant differences were found (all P > 0.05).

**Table 4:** Comparison of U1_SN, U1_NA, IMPA, and L1_NB between groups

Variable (degree)	Group	Median	Mean ± SD	IQR	Min	Max	P-value
		(degree)	(degree)	(degree)	(degree)	(degree)	
T0 U1_SN	without black triangle	101.2	101.08 ± 8.13	12.4	83.9	118.7	0.890
T0 U1_SN	with black triangle	101.0	102.02 ± 10.50	11.0	80.9	133.2	
T1 U1_SN	without black triangle	96.2	97.43 ± 7.20	9.7	83.6	114.7	0.422
T1 U1_SN	with black triangle	97.3	95.36 ± 9.86	12.6	73.5	115.2	
∆ U1_SN	without black triangle	-2.5	-3.65 ± 7.66	10	-21	10	0.282
∆ U1_SN	with black triangle	-5.4	-6.66 ± 10.80	13	-27	19	
T0 U1_NA	without black triangle	3.90	4.56 ± 3.69	4.2	0.3	14.3	0.685
T0 U1_NA	with black triangle	4.60	4.56 ± 3.72	3.4	-3.8	12.9	
T1 U1_NA	without black triangle	3.60	3.57 ± 2.95	5.0	-2.0	9.7	0.106
T1 U1_NA	with black triangle	1.91	1.91 ± 3.81	4.3	-9.1	10.1	
∆ U1_NA	without black triangle	-0.70	-0.99 ± 4.01	4	-11	9	0.159
∆ U1_NA	with black triangle	-3.30	-2.56 ± 3.94	6	-12	5	
T0 IMPA	without black triangle	91.85	92.87 ± 7.95	12.5	82.0	107.9	0.418
T0 IMPA	with black triangle	94.7	92.81 ± 8.71	7.9	68.8	104.0	
T1 IMPA	without black triangle	92.8	90.07 ± 10.65	14.3	68.5	107.1	0.784
T1 IMPA	with black triangle	90.0	90.81 ± 7.60	10.5	69.0	105.8	
∆ IMPA	without black triangle	-3.6	-2.80 ± 7.56	11	-17	14	0.717
∆ IMPA	with black triangle	-2.7	-2.00 ± 7.44	7	-14	20	
T0 L1_NB	without black triangle	5.30	5.43 ± 1.98	2.0	1.3	9.3	0.398
T0 L1_NB	with black triangle	6.50	6.10 ± 3.28	4.7	-1.1	11.1	
T1 L1_NB	without black triangle	4.40	4.56 ± 2.68	2.9	0.0	10.1	0.996
T1 L1_NB	with black triangle	4.70	4.56 ± 2.44	3.1	-0.9	8.4	
∆ L1_NB	without black triangle	-0.90	-0.87 ± 1.97	3	-5	3	0.301
∆ L1_NB	with black triangle	-1.80	-1.54 ± 2.37	2	-6	5	

Table 5 presents vertical incisor movement (U1-PP and L1-MP), crown shape ratios, contact point to alveolar crest distance, adjacent CEJ distance, and angulation changes. Significant differences were found for contact point distance in the lower jaw (P=0.006) and both jaws combined (P=0.003). All other comparisons were not significant (P>0.05).

**Table 5:** Comparison of incisor vertical movements, crown shape, contact point to alveolar crest, CEJ distance

Variable	Group	Median	Mean ± SD	IQR	Min	Max	P-value
T1 U1-PP (mm)	without black triangle	26.85	26.41 ± 2.86	4.10	21.80	32.80	0.75
T1 U1-PP (mm)	with black triangle	28.20	27.20 ± 5.86	3.05	1.42	32.60	
T1 L1-MP (mm)	without black triangle	38.80	39.60 ± 3.56	6.4	34.9	45.2	0.55
T1 L1-MP (mm)	with black triangle	39.2	38.80 ± 8.52	5.8	1.4	47.0	
Crown shape upper jaw	without black triangle	1.27	1.27 ± 0.07	0.08	1.14	1.46	0.197
Crown shape upper jaw	with black triangle	1.30	1.30 ± 0.06	0.11	1.18	1.43	
Crown shape lower jaw	without black triangle	1.19	1.20 ± 0.07	0.09	1.05	1.35	0.053
Crown shape lower jaw	with black triangle	1.23	1.27 ± 0.09	0.07	1.09	1.38	
Contact point to alveolar crest upper jaw (mm)	without black triangle	3.74	4.03 ± 0.73	1.02	2.94	5.64	0.127
Contact point to alveolar crest upper jaw (mm)	with black triangle	4.46	4.42 ± 0.92	1.47	2.58	6.04	
Contact point to alveolar crest lower jaw (mm)	without black triangle	3.20	3.25 ± 0.99	1.91	1.84	5.22	0.006
Contact point to alveolar crest lower jaw (mm)	with black triangle	4.04	4.19 ± 1.00	1.17	2.88	7.42	
Contact point to alveolar crest total (mm)	without black triangle	3.73	3.64 ± 0.75	1.16	2.39	5.43	0.003
Contact point to alveolar crest total (mm)	without black triangle	4.24	4.30 ± 0.69	1.02	3.07	5.68	
CEJ distance upper jaw(mm)	with black triangle	1.58	1.69 ± 0.45	0.69	0.80	2.74	0.169
CEJ distance upper jaw(mm)	without black triangle	2.04	1.88 ± 0.50	0.89	1.02	2.70	
CEJ distance lower jaw(mm)	with black triangle	1.33	1.40 ± 0.43	0.45	0.68	2.66	0.179
CEJ distance lower jaw(mm)	without black triangle	1.50	1.57 ± 0.42	0.62	0.98	2.88	

Table 6 shows changes in Little’s irregularity index. No significant differences were found (P>0.05).

**Table 6:** Changes in Little’s irregularity index

Variable(mm)	Group	Median	Mean ± SD	IQR	Min	Max	P-value
Little’s index upper jaw	without black triangle	14.45	15.92 ± 5.94	8.0	8.8	30.5	0.103
Little’s index upper jaw	with black triangle	12.00	13.25 ± 4.34	5.6	6.3	24.6	
Little’s index lower jaw	without black triangle	10.10	10.87 ± 4.01	6.4	4.5	19.1	0.357
Little’s index lower jaw	with black triangle	12.6	11.9 ± 12.60	5.5	5.8	18.7	
Little’s index combined	without black triangle	12.50	13.86 ± 13.40	6.05	7.85	21.25	0.425
Little’s index combined	with black triangle	11.95	13.57 ± 12.57	4.60	8.15	19.00	

Table 7 shows the comparison of the mean distance between the alveolar bone crest and the contact point before and after treatment. In patients without black triangles, the mean distance in the maxilla and mandible decreased slightly and there is no significant difference between before and after treatment in both jaws (p <0.05). However, in patients with black triangles, the mean maxillary distance changed from 4.32 ± 0.82 mm to 4.42 ± 0.92 mm (p = 0.88), whereas in the mandible it increased significantly from 3.44 ± 0.98 mm to 4.19 ± 1.00 mm (p = 0.021). Thus, a statistically significant change was observed only in the mandibular region of patients with black triangles.

**Table 7:** Comparison of the mean distance between alveolar bone crest and contact point before and after treatment in patients with and without black triangle

Group	Variable	Before treatment (Mean ± SD, mm)	After treatment (Mean ± SD, mm)	P-value
Without black triangle	Maxilla	4.33 ± 0.89	4.03 ± 0.73	0.24 *
	Mandible	3.59 ± 0.86	3.25 ± 0.99	0.22 *
With black triangle	Maxilla	4.32 ± 0.82	4.42 ± 0.92	0.88 #
	Mandible	3.44 ± 0.98	4.19 ± 1.00	0.021#

* = Parametric t-test

# = Wilcoxon signed-rank test (non-parametric)

## DISCUSSION 

Preventing undesirable periodontal changes following orthodontic treatment remains a major clinical concern, particularly the formation of black triangles or open gingival embrasures ^18^^-^^20^. These spaces, resulting from incomplete papilla fill between adjacent teeth, not only affect smile aesthetics but may also compromise oral function. Studies in adult populations report black triangle prevalence ranging from 36% to 42% after orthodontic therapy ^21^. Understanding the contributing factors is crucial for effective prevention and management. The present study aimed to identify risk factors associated with black triangle formation and determine the primary predictor among orthodontic patients.

The variables evaluated included incisor retraction or protraction, intrusion or extrusion, contact point-to-alveolar crest distance, interproximal CEJ distances, axial inclination changes, Little’s Irregularity Index, contact area, and treatment duration. Measurements were performed using standardized panoramic radiographs calibrated with digital calipers, and ImageJ software ensured precise assessment of interproximal distances.

Results revealed a significant increase in contact point-to-alveolar crest distance in patients exhibiting black triangles, both overall and specifically in the mandible. The highest prevalence occurred in the maxillary midline and between the mandibular left central and lateral incisors. In contrast, variables including incisor angulation, vertical movements, CEJ distances, axial inclination changes, Little’s Index, contact area, and treatment duration did not differ significantly between patients with and without black triangles. These findings suggest that vertical interproximal distance, particularly contact point-to-crest spacing, is the most influential factor in post-orthodontic black triangle formation ^22^^-^^24^.

These results align with previous studies. Kim et al. (2018) retrospectively assessed 100 patients and found that excessive contact-to-crest distance, lingual movement of anterior teeth, and anterior-posterior tooth overlap contributed to black triangle formation, while other factors such as treatment duration and incisor angulation changes were statistically non-significant ^23^. In the present study, lingual incisor movement did not show a significant effect, likely due to the controlled sample and uniform extraction protocol.

Jung et al. (2024) evaluated 97 patients, including adults and adolescents, and reported that age, premolar extraction, and upper central incisor angulation changes influenced black triangle occurrence. In the present study, a homogeneous adult population under a standardized extraction protocol minimized these confounding variables, which may explain differences in observed risk factors ^11^.

Periodontal research reinforces the importance of contact point-to-crest distance. Mahasneh et al. (2023) analyzed 404 interproximal sites and demonstrated that a distance of ≤5 mm preserved papilla in 87.5% of cases, whereas distances ≥6 mm substantially increased the risk of papilla loss ^24^. Sghaireen et al. (2015) reviewed 51 studies and concluded that contact-to-crest distance is the principal determinant of black triangle formation, while factors such as tooth morphology, root angulation, periodontal disease, aging, and mechanical trauma play secondary roles ^16^.

Recent CBCT studies by Vandeweghe et al. (2025) confirmed a critical threshold of approximately 4.5 mm for contact-to-crest distance, beyond which black triangle formation becomes more likely. Their study, conducted on patients seeking aesthetic or implant treatment, demonstrated that contact-to-crest distance was a stronger predictor of papilla presence than horizontal crest width or inter-root spacing ^25^. Such findings are consistent with the present study, highlighting the universal importance of vertical interproximal dimension, regardless of treatment modality or patient population.

Contrary to previous reports emphasizing root angulation, crown shape, and contact area as influential factors ^2^^,^^17^^,^^18^, these variables were not significant in the present study. This discrepancy may be explained by the standardized sample, evaluation of six anterior teeth rather than only central incisors, and precise calibration using ImageJ and digital calipers. Moreover, consistent treatment protocols reduced variability, allowing a clearer assessment of primary predictors.

In present study, the distance between the alveolar bone crest and the contact point was assessed to investigate its potential association with the occurrence of black triangles following orthodontic treatment. The findings revealed that in patients without black triangles, the distance showed a slight but statistically insignificant reduction in both arches after treatment. Conversely, among patients who developed black triangles, no meaningful change was observed in the maxillary region, whereas a significant increase in the mandibular distance was detected post-treatment.

The notable increase in the mandibular alveolar bone crest and contact point distance may be explained by both anatomical and biomechanical characteristics inherent to the mandibular anterior region. This area typically exhibits a thinner labial bone plate, narrower interdental spaces, and a delicate gingival biotype, which collectively predispose it to greater crestal bone resorption during orthodontic tooth movement. Additionally, tooth movements such as proclination, retraction, or interproximal enamel reduction can intensify alveolar bone remodeling and contribute to root divergence, ultimately resulting in apical migration of the bone crest. These factors together may elucidate the higher susceptibility of the mandibular region to the development of black triangles.

Despite these meaningful findings, several limitations should be acknowledged. The relatively small sample size (47 patients) may have limited the statistical power and generalizability of the results. The strict inclusion criteria, incomplete records, and time constraints further contributed to this limitation. Potential selection bias is also a concern, as only cases with complete records were included, which may not accurately represent the broader orthodontic population. Moreover, the retrospective nature of the study necessitated the use of panoramic radiographs (OPG) rather than cone-beam computed tomography (CBCT), potentially introducing measurement errors. Although all assessments were performed by a trained examiner, the involvement of a single observer raises the possibility of observer bias. Finally, the study focused solely on the presence or absence of black triangles without evaluating their severity, thereby limiting the clinical depth and interpretive scope of the findings.

Future research with larger, prospective samples and the use of three-dimensional imaging modalities such as CBCT is warranted to validate these findings and to explore the influence of treatment mechanics and periodontal phenotype on post-orthodontic interdental papilla morphology.

Despite the robust methodology, this study has limitations. The sample size was relatively small (n = 47), limiting statistical power and generalizability. Selection bias may exist due to inclusion of only patients with complete records and strict eligibility criteria. Panoramic radiographs, while standardized, may have lower precision compared to CBCT, potentially introducing measurement errors. Observer bias is another potential limitation, although trained examiners conducted all measurements. Furthermore, the study did not categorize black triangle severity, restricting analysis to presence or absence only.

Nonetheless, the study offers valuable insights. It demonstrates that among multiple potential factors, contact point-to-crest distance is the most critical determinant of black triangle formation after orthodontic treatment. Awareness of this factor allows clinicians to plan treatments that preserve papilla integrity, minimize aesthetic compromise, and improve long-term patient satisfaction. Future research should evaluate the influence of different orthodontic systems, wire sequencing, force applications, and treatment timing on black triangle development. Additionally, prospective studies with larger samples can assess intervention efficacy for prevention and management of post-orthodontic black triangles.

In conclusion, maintaining minimal vertical distance between the contact point and alveolar crest is essential in preventing black triangle formation. Other variables, including incisor angulation, crown morphology, axial inclination, and treatment duration, appear less influential under standardized treatment conditions. Integrating these findings into treatment planning can optimize aesthetic outcomes, support interdisciplinary management, and reduce the risk of undesirable periodontal changes post-orthodontics.

## CONCLUSION

Maintaining a minimal vertical distance between the contact point and alveolar bone crest is key to preventing black triangle formation. Under standardized treatment protocols, factors such as incisor angulation, crown morphology, axial inclination, and treatment duration appear less influential. The present study identified a significant post-treatment increase in the mandibular alveolar bone crest to contact point distance among patients who developed black triangles. Incorporating this knowledge into orthodontic treatment planning can enhance aesthetic outcomes, support interdisciplinary care, and reduce the risk of adverse periodontal changes.
